# Physicians’ perceptions of their knowledge and the preparedness of health facilities in Angola to diagnose and manage COVID-19

**DOI:** 10.1093/inthealth/ihab017

**Published:** 2021-04-12

**Authors:** Margarete Arrais, Welwitschia Dias, Jorge M R Gama, Miguel Brito

**Affiliations:** Department of Pulmonology , Military Hospital, Luanda, Angola; CISA – Health Research Centre of Angola, Caxito, Bengo, Angola; Department of Pulmonology , Military Hospital, Luanda, Angola; Centre of Mathematics and Applications, Faculty of Sciences, University of Beira Interior, Covilhã, Portugal; CISA – Health Research Centre of Angola, Caxito, Bengo, Angola; Health and Technology Research Centre (H&TRC), Escola Superior de Tecnologia da Saúde de Lisboa, Instituto Politécnico de Lisboa, Portugal

**Keywords:** Angola, COVID-19, healthcare units, knowledge, physicians

## Abstract

**Background:**

Healthcare professionals represent a vulnerable group in terms of responding to COVID-19. Knowledge can influence healthcare professionals through adoption of the correct attitudes and practices. The aim of this study was to evaluate, by a questionnaire, the perceptions of physicians about their level of knowledge as well as conditions in their workplaces for dealing with COVID-19.

**Methods:**

A cross-sectional study of Angolan physicians took place from 11 May to 23 June 2020. A questionnaire was electronically shared across social media and via email.

**Results:**

The sample consisted of 637 valid questionnaires; 53% of respondents were female, 41% were aged 31–40 y and 79% were from Luanda province. About 51% of physicians perceived that they had adequate knowledge about COVID-19 and 64% used personal protective equipment. These figures were higher among specialists from the province of Luanda. About 81% stated that their health units lacked the technical capacity to diagnose COVID-19. Only 35% of health units have chest tomography equipment; 44% are prepared for the care and/or isolation of patients. Only 33% of units are running intensive care units. The main concerns of physicians were training opportunities and limited access to personal protective equipment.

**Conclusion:**

The results show that it is necessary to strengthen physicians’ knowledge about COVID-19, as well as to improve the conditions of the health units, so as to promote safe practices.

## Introduction

Coronavirus disease 2019 (COVID-19) is an infectious, highly contagious disease caused by severe acute respiratory syndrome coronavirus 2 (SARS-CoV-2).^1,2^ SARS-CoV-2 was first identified in humans in December 2019 in Wuhan, the capital of Hubei Province in China.^1^ This initial outbreak led to a pandemic being declared by the WHO on 11 March, and which has caused an exponential number of cases and deaths worldwide, including in African countries.^1,3^

Health systems in low- and middle-income countries (LMICs) face major challenges in dealing with COVID-19 due to the high pre-existing vulnerability of their health infrastructures and limited numbers of trained healthcare professionals, combined with the diversion of essential medical resources to provide care and management of suspected and sick cases.^4,5^

While hospitals remain under great pressure during this pandemic, it is essential to take immediate measures to contain and mitigate the progression of the disease. These measures aim to identify and isolate early cases, reduce the number of patients at the hospital level, overcome communication and education barriers, as well as to protect patients and healthcare professionals.^6,7^

Healthcare professionals represent a vulnerable group in the response to COVID-19, especially when, for various reasons, biosafety measures are not implemented through personal and collective protection equipment.^8^ This fact has been observed even in countries with well-structured, organised and resourced health systems.^9^ Furthermore, knowledge about a particular pathology can influence healthcare professionals to correct attitudes and practices because incorrect attitudes and practices directly increase the risk of contamination and/or spread of the disease in the case of COVID-19.^9–11^

A statement from the WHO in July 2020 (https://www.afro.who.int/news/over-10-000-health-workers-africa-infected-covid-19, accessed 8 April 2020) declared that about 10% of cases worldwide were among healthcare professionals, although the numbers varied widely among countries.^12,13^ On the same date, the WHO also advised about the threat posed to the African continent and referred to the existence of more than 10 000 infected professionals in 40 countries.^12^

Thus, there is a consensus that the level of knowledge of healthcare professionals and their understanding of the real working conditions in their institutions in the face of this pandemic, in addition to other measures, may also allow for the development and implementation of strategies designed to contribute to its better control.

In Angola, the first two imported cases were reported on 21 March 2020 and, to date, there has been an increasing number of cases, with the epicentre being Luanda province,^14^ which includes the capital of Angola. Luanda is the Angolan province with the largest number of inhabitants, accounting for about 27% of the country's population,^15^ in addition to registering large migratory movements at its points of entry, mainly by air, of people from countries with high incidences of infection.^12,16^

In view of the need to adopt measures to contain the pandemic, a National State of Emergency was decreed on 27 March, and, to date, Angola has been implementing measures to strengthen the National Health Service and is thus approaching the fight against COVID-19 as a major challenge.^14^

Meanwhile, up until August 2020, Angola was operating 22 quarantine centres and 13 treatment centres, distributed nationally but with a greater prevalence in Luanda.^14,17^

The aim of this study was to evaluate the perceptions of Angolan physicians about their level of knowledge, level of preparation and their general workplace conditions for the care of patients with COVID-19.

## Methods

### Study area

The current study took place in Angola, a country located in sub-Saharan Africa with an estimated population of about 30 000 000 inhabitants, of whom about 51% are female.^15^ The country consists of 18 provinces, with Luanda, its capital. It also constitutes the province with the largest number of health units and with the greatest concentration of physicians, even although the ratio of physicians per 10 000 inhabitants stands at 2.38 in Angola.^18,19^

### Study design

This was a cross-sectional study, carried out over 6 wk, from 11 May to 23 June 2020, thus after the emergence of the first imported cases of COVID-19 and involving physicians from public and private health units across Angola.

### Sample size determination

The sample size was calculated according to the formula n≥0.25Nz^2^/((N-1)E^2^+0.25z^2^).^20^ Based on the total number of physicians registered by the Angolan Medical Association (Ordem dos Médicos de Angola) in 2018 (N=6500), with a 95% confidence level (z=1.96) and a margin of error of ≤4% (E=0.04), the estimated sample amounted to 550 physicians.

The inclusion criteria were to be a doctor practising at an Angolan hospital and registered with the Angolan Medical Association. In that sense, all registered doctors were invited to take part. The exclusion criteria were being a physician but not currently engaged in professional practice, returning a questionnaire with incomplete answers or refusing to participate in the study.

### Data collection

This was a descriptive, web-based study following the CHERRIES guidelines.^21^ The data were collected through a self-developed questionnaire according to Google Docs (https://docs.google.com). The online link was shared across various social networking groups such as WhatsApp and was also sent to the email addresses of physicians, who thereby received an invitation to participate. The questionnaire contained a brief introduction, detailed the purpose of the study and the voluntary nature of participation, as well as the guarantee over anonymity and confidentiality. The respective questions spanned (i) sociodemographic data, (ii) information on basic COVID-19 related knowledge perception, namely, the questions: ‘Do you feel you have adequate knowledge about COVID-19?’; ‘Do you feel you have adequate knowledge about the indications and the sample collection methodology for diagnosis of COVID-19?’; ‘Do you consider it essential to perform chest CT for patients with COVID-19?’; and ‘Do you feel you have adequate knowledge about using personal protective equipment (PPE) across its different levels?’; (iii) participation in COVID-19 training sessions; (iv) the existence of working protocols in the institution; (v) the existence of specific areas for patient care with COVID-19; (vi) the existence of means of laboratory diagnosis in their workplaces; as well as (vii) the existence of chest radiography and tomography in their workplaces.

### Statistical methods

Data were processed and analysed with the statistical program IBM SPSS version 26. Categorical variables were described as frequencies and percentages. The variable age was described as mean and SD but in statistical analysis was used as a categorised variable in the following age groups: ≤30, 31–40, 41–50, 51–60 and >60 y.

The associations between each of the variables of interest, ‘health units province’ and ‘adequate knowledge perception’, and other variables through recourse were established by the χ^2^ test or logistic regression, respectively. Also, their respective unadjusted ORs (uORs) were estimated. The calculation of the adjusted ORs (aORs) for the ‘adequate knowledge perception’ variable took into account gender, age and ‘medical training’ and a stepwise selection variables method, based on the likelihood ratio, was then applied (significance level at 5% for a variable entering and 10% for its removal); p<0.05 was considered as statistically significant.

## Results

### General sample characteristics

A total of 704 participants answered the questionnaire, of whom 67 were excluded because they did not meet the inclusion criteria (incomplete questionnaires). Thus, a total of 637 valid questionnaires was obtained.

**Figure 1.**
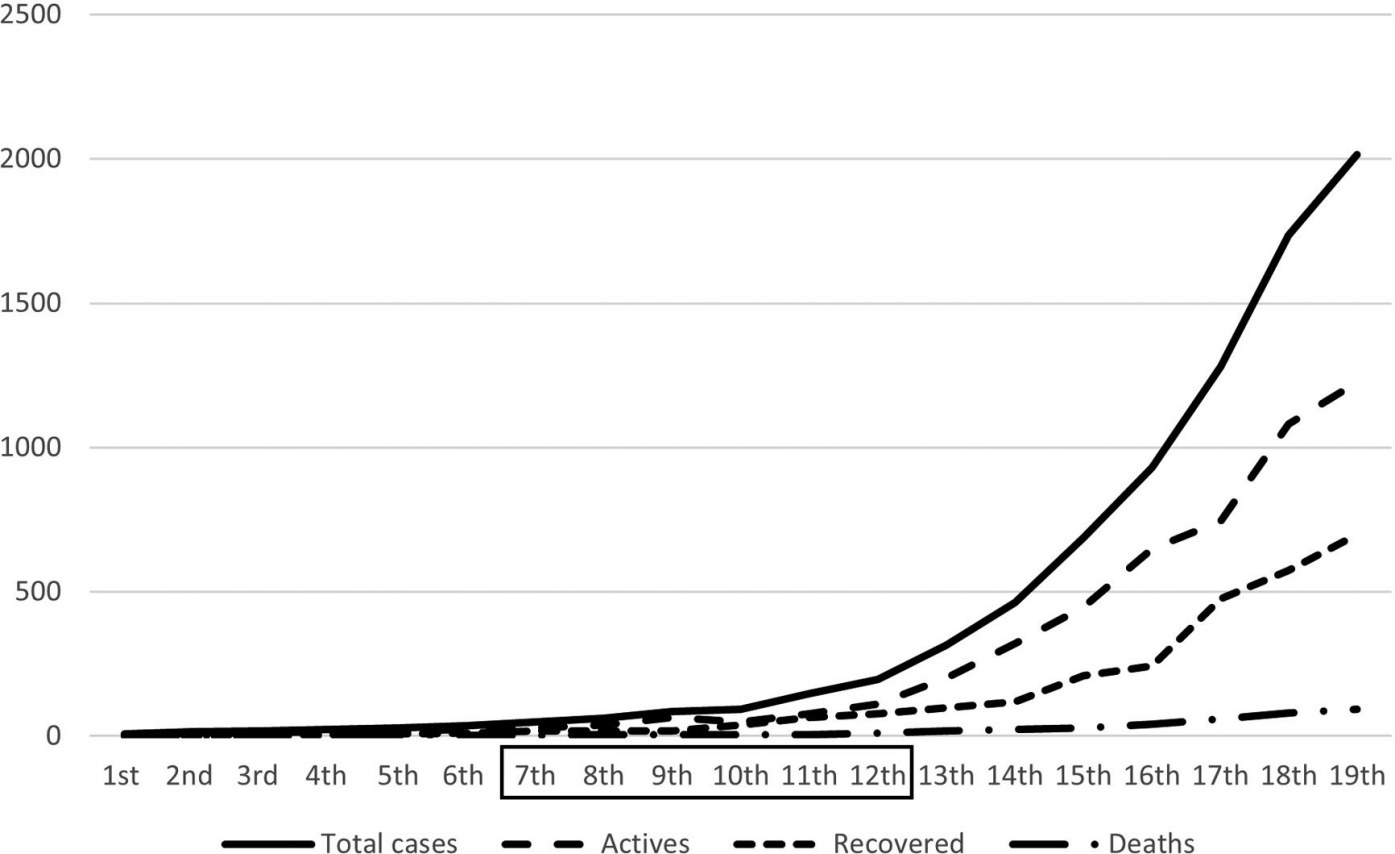
Trends of cases, deaths and recoveries by epidemiological week. (Note that the study period is represented within the box). Data from the Angolan Ministry of Health.^14^

The data collection took place between the 7th and 12th weeks of the pandemic (6 wk), when a significant increase in the number of cases began to be recorded (Figure 1).^14^

The findings report that most participants were female, in the 31–40 y age group, with the professional category of resident or general practitioner and working in the province of Luanda. About 72% of physicians performed their main professional activities in public health units (Table 1).

**Table 1.** General characteristics of the sample (N=637)

Variable	N (%)
Gender	
Female	339 (53.2)
Male	298 (46.8)
Age range (y)	
≤30	142 (22.3)
31–40	260 (40.8)
41–50	115 (18.1)
51–60	93 (14.6)
>60	27 (4.2)
Age Mean±SD	38.77±11.09
Professional category	
Specialist	265 (41.6)
Resident	157 (24.6)
General practitioner	215 (33.8)
Province	
Luanda	505 (79.3)
Other province	132 (20.7)
Health unit	
Public	456 (71.6)
Private	181 (28.4)

### Perception of the knowledge level in relation to COVID-19

The assessment of the perception of the knowledge level incorporated the affirmative answer from physicians of ‘yes’ about COVID-19 based on the questions mentioned in the methodology. This correspondingly found that about 51% of physicians perceived that they had adequate knowledge about COVID-19 and 57% of physicians about the indications and methodology for diagnosis sample collection. Furthermore, 69.7% answered that it is essential to perform a chest tomography on patients with COVID-19.

Regarding PPE, 64.2% of physicians answered that they felt they had adequate knowledge about the measures to be taken according to the various levels of action. There were no statistically significant associations between the perception of the knowledge level and gender, age group, public or private health unit. It was also observed that the proportion of specialist physicians from Luanda province who answered ‘yes’ to the above questions was significantly higher than in other categories of physician (aOR 1.535, 95% CI 1.043 to 2.259; p=0.030) or in other provinces (aOR 2.078, 95% CI 1.178 to 3.666; p= 0.012) (Tables 2 and 3).

**Table 2.** Perception about the level of knowledge (univariate logistic regression). The perception about the level of knowledge was assessed by the following questions: (i) Do you feel you have adequate knowledge about COVID-19?; (ii) Do you feel you have adequate knowledge about the indications and the sample collection methodology for diagnosis of COVID-19?; (iii) Do you consider it essential to perform chest CT for patients with COVID-19?; and (iv) Do you feel you have adequate knowledge about using personal protective equipment across its different levels?

Variable	Adequate knowledge perception		uOR (95% CI)	p-value^a^
N (%)	Yes	No		
	n=136	n=501		
Gender				
Female	67 (19.8)	272 (80.2)	1	0.298
Male	69 (23.2)	229 (76.8)	1.223 (0.837 to 1.788)	
Age range (y)				
≤30	26 (18.3)	116 (81.7)	1	0.852
31–40	58 (22.3)	202 (77.7)	1.281 (0.765 to 2.146)	0.347
41–50	24 (20.9)	91 (79.1)	1.177 (0.634 to 2.185)	0.606
51–60	21 (22.6)	72 (77.4)	1.301 (0.682 to 2.482)	0.424
>60	7 (25.9)	20 (74.1)	1.562 (0.598 to 4.079)	0.363
Professional category				
Specialist	70 (26.4)	195 (73.6)	1.664 (1.137 to 2.437)	0.009
Resident or general practitioner	66 (17.7)	306 (82.3)	1	
Province				
Luanda	120 (23.8)	385 (76.2)	2.260 (1.289 to 3.962)	0.004
Other	16 (12.1)	116 (87.9)	1	
Public health unit				
Yes	95 (20.9)	359 (79.1)	1	0.680
No	41 (22.4)	142 (77.6)	1.091 (0.721 to 1.652)	
Private health unit				
Yes	44 (21.6)	160 (78.4)	1.019 (0.680 to 1.529)	0.926
No	92 (21.2)	341 (78.8)	1	
Training classes				
Yes	113 (22.8)	382 (77.2)	1.531 (0.935 to 2.507)	0.091
No	23 (16.2)	119 (83.8)	1	

Abbreviation: uOR, unadjusted OR.

a Wald test.

**Table 3.** Adjusted ORs ratio of perceptions about the level of knowledge (multivariable logistic regression)

Variable	Adequate knowledge perception		aOR (95% CI)	p-value^a^
N (%)	Yes	No		
	N=136	N=501		
Professional category				
Specialist	70 (51.5)	195 (38.9)	1.535 (1.043 to 2.259)	0.030
Resident or general practitioner	66 (48.5)	306 (61.1)	1	
Province				
Luanda	120 (88.2)	385 (76.8)	2.078 (1.178 to 3.666)	0.012
Other	16 (11.8)	116 (23.2)	1	

Abbreviation: aOR, adjusted OR for age, gender and training.

Likelihood ratio test: p=0.001; Hosmer-Lemeshow test: p=0.711.

a Wald test.

Of these physicians, 21.5% had received training in COVID-19 before the emergence of the first imported cases in Angola; and 55.1% received training in COVID-19 afterwards. About 69% underwent training on the use of PPE for the different levels of contact, even although there was no significant association between the perception of knowledge and whether having undertaken training or not (Table 2).

### Perception of the preparation and general health unit conditions

Regarding perceptions of the level of preparation of their health units, 81% of physicians stated that their units were not able to make any laboratory diagnoses of COVID-19, 35.3% reported that there was the technical capacity to perform radiography and chest tomography, while 36.1% answered that only radiography could be performed.

Physicians also stated that 70.2% of health units had developed action protocols for approaching patients with COVID-19, although only 44% indicated that their unit was prepared for the care and/or hospitalisation of suspected or confirmed cases of infection. However, of these units, only 32.8% ran an intensive care unit (ICU) or had any such area adapted for providing this care. In relation to health units, the results convey the physicians’ perception that hospitals in Luanda are better prepared in relation to these variables when compared with other provinces, namely, specific protocols (OR 2.215, 95% CI 1.489 to 3.294; p<0.001), specific area (OR 2.083, 95% CI 1.382 to 3.139; p<0.001) and specific ICU (OR 2.602, 95% CI 1.613 to 4.196; p<0.001) for approaching COVID-19. It was also assessed whether there was any association with the opportunities prevailing for training physicians in COVID-19 in Luanda and in the other provinces, but the results did not demonstrate any significant differences between these variables (Table 4).

**Table 4.** Perceptions of physicians about preparation and general conditions of health units in other provinces compared with Luanda (χ^2^ test). The perception about preparation and the general conditions of health units was assessed by the following questions: (i) Did you do any training classes on COVID-19 at your workplace?; (ii) Are there any action protocols in your workplace to deal with COVID-19 patients?; (iii) Is there a specific area in your workplace to treat COVID-19 patients?; and (iv) Is there a specific area (e.g. intensive care unit [ICU]) in your workplace to treat critical COVID-19 patients?

Variable	Health unit province		OR (95% CI)	p-value^a^
N (%)	Luanda	Other		
	N=505	N=132		
Training classes				
Yes	396 (78.4)	99 (75.0)	1.211 (0.774 to 1.894)	0.401
No	109 (21.6)	33 (25.0)	1	
Specific protocol				<0.001
Yes	373 (73.9)	74 (56.1)	2.215 (1.489 to 3.294)	
No	132 (26.1)	58 (43.9)	1	
Specific area				
Yes	240 (47.5)	40 (30.3)	2.083 (1.382 to 3.139)	<0.001
No	265 (52.5)	92 (69.7)	1	
Specific ICU				
Yes	185 (36.6)	24 (18.2)	2.602 (1.613 to 4.196)	<0.001
No	320 (63.4)	108 (81.2)	1	

a χ^2^ test.

At the end of the questionnaire, the respondents had an option to submit a comment should they wish. Of the 637 physicians, comments were provided by a total of 171 (26.8%). Among them, 51 (29.8%) took the opportunity to state they had no had opportunities for COVID-19 related training and/or on personal protection measures at different levels of action, 39 (22.8%) complained about the lack of PPE and 28 (16.4%) about inappropriate workplace conditions, such as specific facilities for isolating and treating suspected cases of COVID-19.

Nevertheless, 63 participants (36.8%) praised the initiative, with some recognising the importance of this type of information for a better perception of the real local conditions to better define strategies, improving the approach to specific cases as well as for the prevention of in-hospital contamination.

## Discussion

This is one of the few studies on physicians, healthcare units and COVID-19 in Africa and the first in Angola. The study took place after the emergence of the first imported cases of COVID-19 in the country, between the 7th and 12th weeks of the pandemic, and it identifies how most physicians perceive that they have adequate knowledge about COVID-19 and the use of PPE, especially among those working in Luanda.

Knowledge is a prerequisite ability to establish beliefs over prevention, define positive attitudes and promote positive behaviours, which can influence healthcare professionals in the effectiveness of their strategies to deal with certain diseases.^9,10^ The results of the current study, carried out only with physicians, reveal that their perception of knowledge levels regarding COVID-19 are comparatively similar to those described in other African countries,^22–24^ but are much lower than those reported in China,^25^ Nepal,^26^ Vietnam^27^ and Lebanon,^28^ although in most of these studies, not only physicians, but also other healthcare professionals participated, including nurses, paramedics, diagnostic technicians and other health technicians. In addition, those other studies applied measurement scores and their questionnaires addressed not only the level of knowledge, but also attitudes and practices. These findings clearly emphasise a requirement to improve levels of knowledge about COVID-19 among Angolan physicians so as to promote safe practices and attitudes, as well as prevention and control measures, even although the present results did not identify any significant differences between adequate knowledge and whether having undergone training or not. The present results also convey how specialised physicians displayed a greater capacity to answer affirmatively, once again emphasising the need for specialised training, as reported in studies from various regions of the world, including the African continent.^27,29,30^

The results state that around 81% of health units are not able to diagnose COVID-19 and that in only 35% is it possible to perform radiography and chest tomography. Protocols for dealing with COVID-19 cases were developed in about 70% of health units, but only 44% of them were prepared for the care and/or hospitalisation of suspected or sick cases; and only about 33% were running ICUs. In relation to these variables, health units in the Luanda province were significantly better prepared than health units in other provinces. There is a gap in LMICs because differentiated medical care, as well as training and opportunities for promotion, are limited and centralised in major cities and urban regions.^31^ African countries need to expand the capacity of their health units, both in human resources and in technical resources, especially for primary care, and to ensure that primary emergency care is included in primary health systems so as not to overburden central health units.^31^

Published data on the availability of intensive care resources in LMICs are scarce. Existing ICUs interlink with the national hospital bed capacity and health expenditure. For example, South Africa and Uganda have about 75 and 1 ICU beds per million population, respectively,^32^ compared with 336 ICU beds per million population in the USA.^33^ Most sub-Saharan African countries have <20 ICU beds for their entire population.^32^

As observed, the difficulties in health units and in supplying PPE are common at this stage of the pandemic in almost every country worldwide, but especially in LMICs,^13,34,35^ and Angola is no exception, as the current study reports. Furthermore, there is a recognition that the lack of biosafety equipment increases the fear and insecurity of professionals caring for patients with COVID-19, in keeping with the high rates of infection and death. A study carried out with healthcare professionals in New York concluded that the scarcity of PPE contributed to 68% of the factors causing distress to healthcare professionals during the pandemic phase, contributing negatively to their mental health and productivity.^36^

Finally, it is important to highlight that a well-functioning health system is built on a number of factors, including qualified, trained and motivated healthcare professionals, adequate infrastructure, as well as acceptable quantities of resources and medicines, including PPE, of a satisfactory quality, duly supported by well-structured and evidence-based health policies.^37^

The current study has several limitations. Because this is a study based on participant responses, which are dependent upon honesty, interpretation and memory capacity, it may have incurred a series of biases. The self-developed questionnaire applied was not validated and it evaluated the perceptions of physicians’ knowledge with no measurement tool applied, which subsequently impaired comparisons with other studies. Despite having obtained national coverage, probably not all physicians had access to the questionnaire, due to various difficulties, such as internet access, especially in the different provinces of Angola. However, the results obtained provide useful data regarding the current levels of preparation of physicians and health units in the country, serving as support for the planning of specific actions to address the COVID-19 pandemic, not only in Luanda, where there is currently an exponential increase in cases, but also in the various provinces of the country, where cases of COVID-19 have mostly already been recorded.

### Conclusion

The current study reports the perceptions that Angolan physicians have regarding their knowledge related to COVID-19 and the preparation of their workplaces to care for patients with COVID-19. The results shows that it is necessary to strengthen knowledge about COVID-19, as well as to improve the conditions of health units, to promote safe practices. The findings of this study are important for the development of appropriate strategies to improve the care of patients, to prevent in-hospital infections, complications and deaths.

### Recommendations

With the rising number of COVID-19 cases in Angola, greater efforts are needed in the training and capacitation of physicians, through recourse to social media networks, improvement of sanitary infrastructures, as well as providing them with diagnostic laboratories and supplying biosafety materials. This strategy will lead to physicians feeling better motivated to treat patients using better practices and with greater safety, ensuring control of this pandemic and the challenges it poses to humanity.

Future studies with a standardised and validated methodology, with the inclusion of different healthcare professionals, in the different provinces of Angola, should be carried out to verify more comprehensively and reliably the aspects that have not here been subject to clarification.

## Data Availability

Data supporting the findings of this study are available from the corresponding author on reasonable request.
